# On-farm broiler chicken welfare assessment using transect sampling reflects environmental inputs and production outcomes

**DOI:** 10.1371/journal.pone.0214070

**Published:** 2019-04-17

**Authors:** Neila BenSassi, Judit Vas, Guro Vasdal, Xavier Averós, Inma Estévez, Ruth C. Newberry

**Affiliations:** 1 Department of Animal Production, Neiker-Tecnalia, Vitoria-Gasteiz, Spain; 2 Department of Animal and Aquacultural Sciences, Faculty of Biosciences, Norwegian University of Life Sciences, Ås, Norway; 3 Norwegian Meat and Poultry Research Centre, Oslo, Norway; 4 Ikerbasque, Basque Foundation for Science, Bilbao, Spain; Tokat Gaziosmanpasa University, TURKEY

## Abstract

To evaluate the utility of transect sampling for assessing animal welfare in large chicken flocks, we quantified relationships between environmental inputs, welfare problems detected using transect sampling, and production outcomes. We hypothesised that environmental inputs including environmental complexity (i.e. number of environmental enrichment types provided), space allowance, underfloor heating (presence or absence), and photoperiod regimen (18 h continuous vs 16 h intermittent) would correspond to variations in welfare assessment findings, which would predict production outcomes. We conducted on-farm welfare assessment of Norwegian broiler flocks at approximately 28 days of age. We sampled four transects (rows between feeder and drinker lines) per flock to determine litter quality and the proportions of chickens with compromised welfare as indicated by visual signs of walking difficulties, illness, skin wounds and small bird size. Production outcome measures included mortality, reasons for carcass rejection at slaughter, footpad dermatitis, growth rate, feed conversion and an integrated production index. Greater environmental complexity was associated with a reduction in skin wounds and total welfare problems on the farm, lower mortality, fewer rejections due to wounds and underweight birds, and fewer rejections overall. Higher space allowances within levels of environmental complexity were associated with fewer walking difficulties and welfare problems overall, a reduction in rejections due to wounds, and a higher growth rate and production index. Underfloor heating was associated with a reduction in rejections due to leg deformity, and intermittent light was associated with lower illness and skin wound rates on the farm, and lower mortality. Furthermore, fewer welfare problems and better litter quality on the farm were associated with fewer carcass rejections at slaughter. Thus, data from transect sampling varied with environmental inputs and production outcomes, supporting the validity of transect sampling for practical, animal-based on-farm welfare assessment.

## Introduction

In modern animal agriculture, animals are kept in large groups, with flocks, schools, and herds numbering in the thousands. Keeping track of the welfare of individual animals in such large groups presents challenges. It is, thus, common to base animal welfare assessment on adherence to engineering standards (i.e. *a priori* resource-based rules such as the provision of a certain amount of space/animal). However, because animal welfare is about quality of life, animal welfare assessment has greater face validity when based on observation of animals than inference based on resource provision (e.g. [1, 2]). Assessment of living animals also has greater face validity than welfare assessment based on *a posteriori* review of animal-based production records and slaughter plant health inspection outcomes. Yet, existing on-farm welfare assessment protocols (e.g. [3]) tend to be time-consuming [2, 4, 5], making them more suited for detailed research investigations than as practical industry management tools. In addition, animals can be stressed by protocols that require them to be handled for close examination of their physical condition, which may affect results [4]. There is a need, therefore, for simple and efficient, low-stress tools for welfare assessment on the farm.

Practical welfare surveillance methods for use in large commercial poultry houses are particularly needed. Poultry kept for meat production can be afflicted by a variety of welfare challenges including wounds, infections, and cardiovascular and skeletal metabolic disorders that compromise normal development [6, 7, 8, 9]. To assess the prevalence of such welfare problems in a practical, low-stress but systematic manner, a transect sampling procedure has been developed [4] and validated against the “gold standard” of evaluating every bird in the flock [10]. According to the transect sampling method, an observer walks slowly along successive transects in the house, defined as rows between lines of feeders and drinkers. In each transect, birds located in the space immediately ahead of the observer are scanned as they move out of the path of the approaching observer. The observer records all birds within the transect that are observed to be clearly afflicted by specific welfare-relevant conditions as identified by the European Food Safety Authority Panel on Animal Health and Welfare [11]. For broiler chickens, these welfare “red flag” indicators include: lame, immobile, head, back and tail wounds, small, dirty, featherless, sick, terminally ill, and dead birds. To optimise surveillance time and minimise the risk of missing or recounting birds, only clearly evident “iceberg” cases that can be rapidly categorised are recorded, rather than attempting to score every bird on a graded scale of severity. Litter quality is also rated at three points along each transect. A free android smartphone application (i-WatchBroiler) has been designed for use in entering the data collected during transect walks in broiler houses [12].

Transect sampling is intended for implementation by stakeholders including farmers, veterinarians, animal welfare auditors and advisors to obtain a quick but quantitative snapshot of the current welfare status of the flock. However, previous research indicates the potential for some variation in results from different observers and sampled transects [4, 10, 13], which could mask differences in welfare between flocks. It has not yet been established whether transect sampling can reveal differences in flock welfare according to environmental conditions (e.g. environmental enrichment, space allowance, heating systems, lighting programmes), or offer a useful forecast of production outcomes.

Environmental enrichment refers to additions and modifications to the housing environment that increase environmental complexity, stimulate species-specific behaviour and facilitate biological functioning, thereby improving animal welfare [14, 15]. Elevated resting surfaces such as perches, platforms and boxes can serve as environmental enrichment for broilers [16]. Use of such structures may strengthen muscles and joints and enhance the blood supply to the legs, wings and heart [17]. Their provision has been associated with greater tibial diaphysis diameter [18], a reduction in tibial dyschondroplasia [19], reduced severity of footpad dermatitis [20], lower mortality due to heat stress [21], and a lower heterophil to lymphocyte ratio [22, 23]. Peat moss is an environmental enrichment material that stimulates ground scratching and dustbathing behaviour [24, 25, 26]. A negative association between lameness score and dustbathing frequency has been reported [27], suggesting that peat could improve leg health. Moreover, peat can have beneficial effects on the digestive tract mucosa [28], and its use as a feed supplement has been associated with increased weight gain, and feed efficiency [29, 30, 31]. Bales of litter material provide enrichment by stimulating exploratory pecking and foraging behaviour as well as acting as platforms [32, 33]. Collectively, increased environmental complexity resulting from provision of multiple types of enrichment simultaneously may have additive welfare benefits.

Space allowance is also of relevance to broiler welfare and production outcomes. In different studies, increasing the space allowance from 0.044–0.083 m^2^/bird [34], 0.046–0.074 m^2^/bird [35], or 0.066–0.500 m^2^/bird [36], reduced the prevalence or severity of footpad dermatitis. An increase in space allowance from 0.044–0.083 m^2^/bird [34], or reduction in the stocking density from 44.8–15.9 kg/m^2^ [37], also improved walking ability. In addition, more space/bird has been associated with lower mortality, contact dermatitis and carcass bruising [38], and increased growth and feed efficiency (e.g. when comparing 30–35 kg/m^2^ with 40 kg/m^2^ [35, 39, 40, 41] and 50 kg/m^2^ [42]).

Underfloor heating is reported to improve temperature uniformity and efficiency of temperature distribution in comparison to hood heating [43]. It may also reduce litter moisture and ammonia, resulting in reduced cardiovascular disease and ascites [44], leg problems [37], footpad dermatitis and feather dirtiness [45]. Underfloor heating has been associated with a reduction in footpad dermatitis in turkeys [46, 47], and with lower mortality, greater weight gain and lower feed consumption in broilers [48].

The photoperiod regimen can affect body temperature and the immune system [49], and regulate feed intake [50] and physical activity [51]. Long photoperiods (20–24 h) have been associated with susceptibility to leg problems in broilers [37, 52] and increased mortality and leg problems in Japanese quail [53]. An intermittent photoperiod regimen alternating between 2 h light and 2 h dark has been associated with less footpad dermatitis and higher body weights in broilers when compared to a short (8 h) continuous photoperiod [54]. Provision of at least 6 h of darkness/day is now required in the European Union, with at least one uninterrupted dark period of at least 4 h [55]. In Norway, the latter provision is limited to two uninterrupted dark periods of at least 4 h [56]. These requirements have resulted in two typical forms of photoperiod regimen in Norway, one comprising 18 h of continuous light and the other having two 4-h dark periods/day (i.e. 16 h intermittent light).

In the current study, we used the transect sampling method to collect data from Norwegian commercial broiler flocks. Our aim was to investigate relationships between environmental factors, transect data and production data (including health inspection findings from the slaughter line where every bird in the flock is evaluated). Based on previous reports, we hypothesised that environmental complexity (defined as the number of environmental enrichment types provided), space allowance, underfloor heating, and photoperiod regimen would be correlated with both transect and production findings. Specifically, we predicted that greater environmental complexity, greater space allowance, presence (vs absence) of underfloor heating, and 16 h intermittent (vs 18 h continuous) lighting would be associated with indications of improved welfare both on the farm and at slaughter. Further, we expected to find positive associations between on-farm welfare problems and causes of rejection at slaughter, and a negative association between litter quality and footpad dermatitis. Additionally, we examined the consistency of transect data between observers, and between transect locations within the house (left vs right side, beside wall vs more central).

## Materials and methods

### Ethics statement

The study was conducted between February and May, 2017, on 15 farms located in southeast Norway. All farms belonged to the same cooperative, and functioned in accordance with Norwegian animal welfare legislation governing poultry production [56]. Farm owners gave their consent to participate in the research, participation was voluntary, and no personal details were collected. No biological samples were collected for research purposes. Because no experimental manipulations were made and observations were non-invasive, the study did not require approval of animal use by the Norwegian Food Safety Authority [57].

### Environmental inputs

At each farm, we evaluated two consecutive Ross 308 mixed sex flocks kept in the same house (Table 1). All houses were well-insulated, with concrete floors and automatic drinkers, feeders and ventilation systems. Ten houses had underfloor heating. Houses were thoroughly cleaned and supplied with a thin layer of fresh litter material (generally softwood shavings) before placement of each flock. Initial flock size ranged from 9,600–34,050 broilers (mean ± SE, 19,480 ± 809) and initial space allowance ranged from 0.056–0.073 m^2^/bird. Artificial lighting was provided by LED lights, with either a single 6-h dark period or two 4-h dark periods daily from 7 days of age until 3 days before slaughter. Farmers checked their flocks at least twice daily. They removed any birds found dead, humanely culled any moribund or severely disabled birds, and kept records on these numbers. On the day of slaughter (between 32 and 35 days of age), the mean stocking density (± SE) was 32.08 ± 0.63 kg/m^2^.

**Table 1:** Environmental provisions to each flock.

Farm	Under-floor heating	Lighting regimen (18 h continuous vs 16 h intermittent)	Flock	Space allowance (m^2^/chick started)	Environmental enrichment type (X indicates provision)		
					Boxes	Peat	Wood shavings bales
1	Yes	18 h	1	0.070	X	X	X
			2	0.061		X	
2	Yes	18 h	1	0.066	X	X	X
			2	0.073	X	X	X
3	No	16 h	1	0.057			
			2	0.073	X	X	X
4	Yes	18 h	1	0.056			
			2	0.072	X	X	X
5	Yes	16 h	1	0.057			
			2	0.072	X	X	X
6	No	18 h	1	0.057	X	X	X
			2	0.072	X	X	X
7	Yes	16 h	1	0.068	X	X	X
			2	0.058			
8	Yes	18 h	1	0.061	X	X	X
			2	0.074	X	X	X
9	Yes	18 h	1	0.063	X		
			2	0.071	X	X	
10	No	16 h	1	0.072	X	X	X
			2	0.060	X	X	
11	No	16 h	1	0.062			
			2	0.071	X	X	X
12	No	18 h	1	0.069	X	X	X
			2	0.063		X	
13	Yes	16 h	1^1^	0.058	X		X
			2	0.060	X		
14	Yes	18 h	1	0.060	X		X
			2	0.066	X		
15	Yes	18 h	1	0.061	X	X	
			2	0.063			

^1^On-farm welfare assessment data missing from this flock.

The cooperative had a recommended environmental enrichment programme, which involved providing boxes for perching by 7 days of age (1 box/50 m^2^), and peat (10 l/50 m^2^) and wood shavings bales (1 half-bale/100 m^2^) at 7, 14, 21 and 28 days of age. Farmers supplied these enrichments to varying degrees in the different flocks (Table 1). The boxes were either cardboard or plastic, ranging from about 0.2–0.25 m high and with an upper surface area of about 0.2–0.3 m^2^. Some boxes had openings allowing birds to go under them when young, and some were stacked in pyramids. The boxes remained in the house throughout rearing. Peat was provided over the whole floor (as litter), loose in piles, contained in low surrounds, or as bales (200 l bales, presented whole or cut in half). Wood shavings bales (25 kg) were cut in half and presented with or without removal of their plastic wrapping. Because the added peat and wood shavings bales became integrated into the litter, they were renewed weekly.

### On-farm welfare assessment

Two trained observers visited each flock once at 26–30 days of age, shortly before slaughter when welfare problems were most evident. Before starting the data collection, we determined the dimensions of the house (mean ± SE, 1284 ± 53 m^2^), and width of each transect (mean ± SE, 2.06 ± 0.04 m). Transects were defined as wall or central transects. Wall transects comprised the area demarcated by a side wall and the nearest feeder or drinker line (whichever was closest to the wall, typically a drinker line), extending the length of the house from one end wall to the other end wall. Central transects comprised the area bounded by adjacent feeder and/or drinker lines (typically one of each), extending the length of the house. Transects were numbered consecutively starting with the wall transect on the left side of the house, as viewed when standing at the end of the house closest to the entrance door.

On farm visits, each observer assessed the prevalence of the welfare indicators (Table 2) within one central transect and one wall transect, for a total of four transects/flock. One observer sampled two transects on the left side of the house, walking up one transect and returning down the other, and the second observer sampled two transects on the right side of the house. While one observer sampled a wall transect, the other simultaneously sampled a central transect, and vice versa. We randomised the side of the house evaluated by each observer, and alternated the order of observing wall and central transects. Both wall transects were included in the flock assessment because observations of Spanish broiler flocks indicated that immobile, small, sick, dirty and/or dead birds were more likely to be found in wall than central transects [4, 13]. We selected the two observed central transects pseudo-randomly, avoiding contiguous transects to minimise double counting of the same birds, and any atypically wide (> 3 m) or narrow (< 1 m) transects. Evaluation of two transects took an average of 30–35 min depending on house length. Birds were assigned to the welfare indicator best describing their condition based on rapid visual assessment.

**Table 2:** Ethogram of broiler welfare problems recorded during transect sampling, and subsequently pooled categories.

Indicator	Description	Category
Lame	Walks with obviously uneven strides or unsteady steps. May exhibit outward or inward twisting of one or both legs leading to severe limping. Lameness is clearly advanced rather than in early stages.	Walking difficulties
Immobile	Does not move away when approached or moves by propping on wings or crawling. If gently nudged, moves with difficulty, no more than three steps before sitting down again.	
Sick	Signs of impaired health, including small and/or pale comb, red, watery or closed eyes, retracted neck and disarranged/raised feathers. Usually found in a resting position. Includes wry neck.	Illness
Terminally ill	Lying with head resting on ground or lying on back, with signs of being close to death (e.g. laboured breathing, half-closed eyes). Excludes panting related to heat stress.	
Dead	No signs of life.	
Head wounds	Skin scratches on head or neck indicated by the presence of fresh or dried blood/scabs visible from 1–2 m away.	Skin wounds
Back wounds	Skin scratches on back (between neck and tail) and/or wings indicated by the presence of fresh or dried blood/scabs visible from 1–2 m away.	
Tail wounds	Skin scratches around tail and hips indicated by the presence of fresh or dried blood/scabs visible from 1–2 m away.	
Small	Stunted growth. Approximately half average size of flock mates. May have yellow downy feathers, especially on head.	Small
Dirty	Extensive dark staining of body sides, wings, chest, back, and/or tail feathers due to prolonged contact with wet litter. Excludes light soiling or discolouration of feathers caused by dust, peat or excrement.	Not observed
Featherless	Lacking feathers on majority of back and wings. Excludes moulting.	Not observed

We observed no dirty or featherless birds in the flocks visited. Due to low numbers, we assigned the transect counts for the remaining indicators to four broader categories: walking difficulties, illness, skin wounds, or small (Table 2). The counts in each welfare category were summed across the four assessed transects and expressed as a proportion of the total number of birds estimated to be present in those four transects. This denominator was calculated based on the total number of birds present in the house on the assessment day and the dimensions of the transects, assuming a uniform distribution of birds across the house. We also calculated an overall welfare problems index (i.e. summed counts across all categories as a proportion of the estimated number of birds in the observed transects). We evaluated litter quality at the beginning, middle, and end of each walked transect on a 5-point scale, from 0 (dry and loose litter) to 4 (caked litter) based on the Welfare Quality [3] protocol for poultry, and calculated the average litter score/flock.

### Production outcomes

Flocks were slaughtered at a mean age of 33.6 days, all at the same slaughter plant following 2-phase CO_2_ gas stunning. Production data on each flock were provided by the farmers and the slaughter plant. We calculated total mortality on the farm up to the day of slaughter as [(found dead + culled)/number of chicks started]. Reasons for carcass rejection were routinely recorded by health inspection personnel stationed along the slaughter line. They recorded the primary reason for rejection of each bird though multiple reasons could exist. We categorised these reasons as: perosis (any pronounced leg deformities), illness (sum of liver disease, heart disease, ascites, persistent egg yolk, and discolouration/suspicious smell), wounds (scratches, bruises, hematomas, fractures and dislocations), and underweight (below marketable weight). We expressed the numbers rejected in each category, and total number rejected, as a proportion of the total number of birds slaughtered. Footpad dermatitis was evaluated by slaughterhouse personnel according to standard procedure for Norwegian flocks, whereby 100 feet/flock were assessed on a 3-point scale (0 = no lesions, 1 = mild lesions, 2 = severe lesions), and points were summed to give a flock score ranging from 0–200. Further flock data included growth rate [mean g eviscerated carcass weight/days of age at slaughter], and the feed conversion ratio [total kg feed provided/((number slaughtered − number rejected) * mean kg eviscerated carcass weight)]. We also calculated an integrated production index value for each flock [mean g eviscerated carcass weight * (number slaughtered − number rejected)/(days of age at slaughter * number of chicks started)].

### Statistical analysis

We analysed all data using SAS 9.4 (SAS Institute, NC, USA). Associations of the four environmental inputs (environmental complexity, space allowance, underfloor heating, and photoperiod regimen) with each on-farm welfare assessment variable and each production outcome variable were investigated using generalised linear mixed models (GLIMMIX procedure). Environmental complexity (i.e. number of environmental enrichment types provided) was treated as a continuous variable ranging from 0–3 to explore linear trends irrespective of the specific combinations of enrichment materials used. This approach recognised the underlying continuity of complexity despite imprecise quantification. Due to collinearity between space allowance and environmental complexity, the residuals of space allowance regressed on environmental complexity were included in the model as a continuous variable describing the variation in space allowance around the regression line at each level of environmental complexity. Underfloor heating (absence vs presence) and photoperiod regimen (18 h continuous vs 16 h intermittent) were categorical factors. We used additional GLIMMIX models to estimate associations of the on-farm welfare assessment variables (1) walking difficulties, illness, skin wounds, and small birds, (2) the welfare problems index, and (3) litter score, with the production outcomes (total mortality, reasons for rejection at slaughter, total rejections, footpad dermatitis score, growth rate, feed conversion ratio, production index). Farm was included as a random effect in all models (see S1 Appendix for model specification details and covariance estimates for farm).

Response variables comprising counts expressed as proportions were analysed according to the binomial distribution with logit link, maximum likelihood estimation and Laplace likelihood approximation. Because flock footpad dermatitis scores were heavily right-skewed, with a majority of flocks receiving a score of 1, we compared flocks receiving scores of 1 vs > 1 based on the binary distribution with logit link. We analysed the remaining response variables (mean litter score, growth rate, feed conversion, production index) according to the gamma distribution with log link and residual pseudo-likelihood estimation (see S1 Appendix for details). We applied the inverse link to back-transform estimated values (continuous factors) and least squares means (categorical factors) to their original scale for graphical presentation. The absence of underfloor heating, and a continuous photoperiod of 18 h, served as the reference levels for least squares means estimation.

We also evaluated the degree of agreement in findings on the proportion of birds with walking difficulties, illness, skin wounds, and small birds between pairs of transects within flocks. We compared the differences between the pairs of transects that were (1) assessed by different observers, (2) located on the left vs right side of the house and (3) located in wall vs central transects, using the Wilcoxon signed-ranks test in the UNIVARIATE procedure.

## Results

### Descriptive data

The welfare problem indicators occurred at low levels (Table 3; S1 Appendix), with lameness contributing most to the welfare problems index. Skin wounds were most common in the tail region. The litter scores at sampled locations varied from 0–2, with no scores of 3 or 4 being recorded. Culling by the farmer accounted for about 30% of the mortality. Ascites and liver disease were the most common reasons for rejection at slaughter, resulting in the pooled illness category accounting for the majority of rejections. Footpad dermatitis occurred at low levels, with flock scores ranging from 1–13 of a possible 200.

**Table 3:** Prevalence of welfare problems detected by transect sampling during on-farm welfare assessment, and production outcomes.

Welfare indicator^1^	Mean	SE	Production outcome^2^	Mean	SE
Lame (%)	0.22	0.02	Mortality (%)^3^	3.59	0.35
Immobile (%)	0.07	0.01	Culled (% of mortality)^4^	30.31	3.91
Walking difficulties (%)^5^	0.29	0.03	Rejection due to perosis (%)	0.01	<0.01
Sick (%)	0.03	<0.01	Rejection due to liver disease (%)	0.35	<0.01
Terminally ill (%)	<0.01	<0.01	Rejection due to heart disease (%)	0.07	0.01
Dead (%)	0.04	0.01	Rejection due to ascites (%)	0.71	0.06
Illness (%)^6^	0.07	0.01	Rejection due to persistent egg yolk (%)	0.01	<0.01
Head wounds (%)	0.01	<0.01	Rejection due to discolouration/smell (%)	0.05	0.01
Back wounds (%)	<0.01	<0.01	Rejection due to illness (%)^7^	1.19	0.11
Tail wounds (%)	0.08	0.01	Rejection due to wounds (%)	0.14	0.04
Skin wounds (%)^8^	0.08	0.01	Rejection because underweight (%)	0.20	0.08
Small (%)	0.12	0.04	Total rejections (%)^9^	1.66	0.15
Dirty (%)	0	0	Footpad score^10^	2.37	0.46
Featherless (%)	0	0	Growth rate (g/day)^11^	43.20	0.42
Welfare problems index (%)^12^	0.57	0.06	Feed conversion (ratio)^13^	2.22	0.02
Litter score^14^	1.17	0.06	Production index (g/day)^15^	40.86	0.42

^1^On-farm welfare indicator data from transect sampling on 15 farms (2 flocks/farm; n = 29 flocks). Counts expressed as % of estimated number of birds in four walked transects per flock.

^2^Flock production data (n = 30 flocks). Reasons for rejection counts expressed as % of total number of birds slaughtered.

^3^[(Found dead + culled)/number of chicks started] up to day of slaughter, expressed as %.

^4^Number culled, as a % of mortality up to day of visit (n = 26 flocks due to missing data).

^5^Includes lame and immobile.

^6^Includes sick, terminally ill and dead.

^7^Includes liver disease, heart disease, ascites, persistent egg yolk and discolouration/suspicious smell.

^8^Includes head, back and tail wounds.

^9^Includes perosis/leg deformity, rejection due to illness, rejection due to wounds and rejection because underweight.

^10^100 feet/flock scored on 3-point scale (0 = no lesions, 1 = mild lesions, 2 = severe lesions), giving a maximum possible flock score of 200.

^11^[Mean g eviscerated carcass weight/days of age at slaughter].

^12^Includes lame, immobile, sick, terminally ill, dead, head, back, and tail wounds, and small.

^13^[Total kg feed provided to flock/((number slaughtered − number rejected) * mean kg eviscerated carcass weight)].

^14^Scored from 0 (dry, loose litter) to 4 (caked litter) in three locations / transect.

^15^[Mean g eviscerated carcass weight * (number slaughtered − number rejected)/(days of age at slaughter * number of chicks started)].

### Associations of environmental inputs with welfare assessment findings and production outcomes

**Fig 1:**
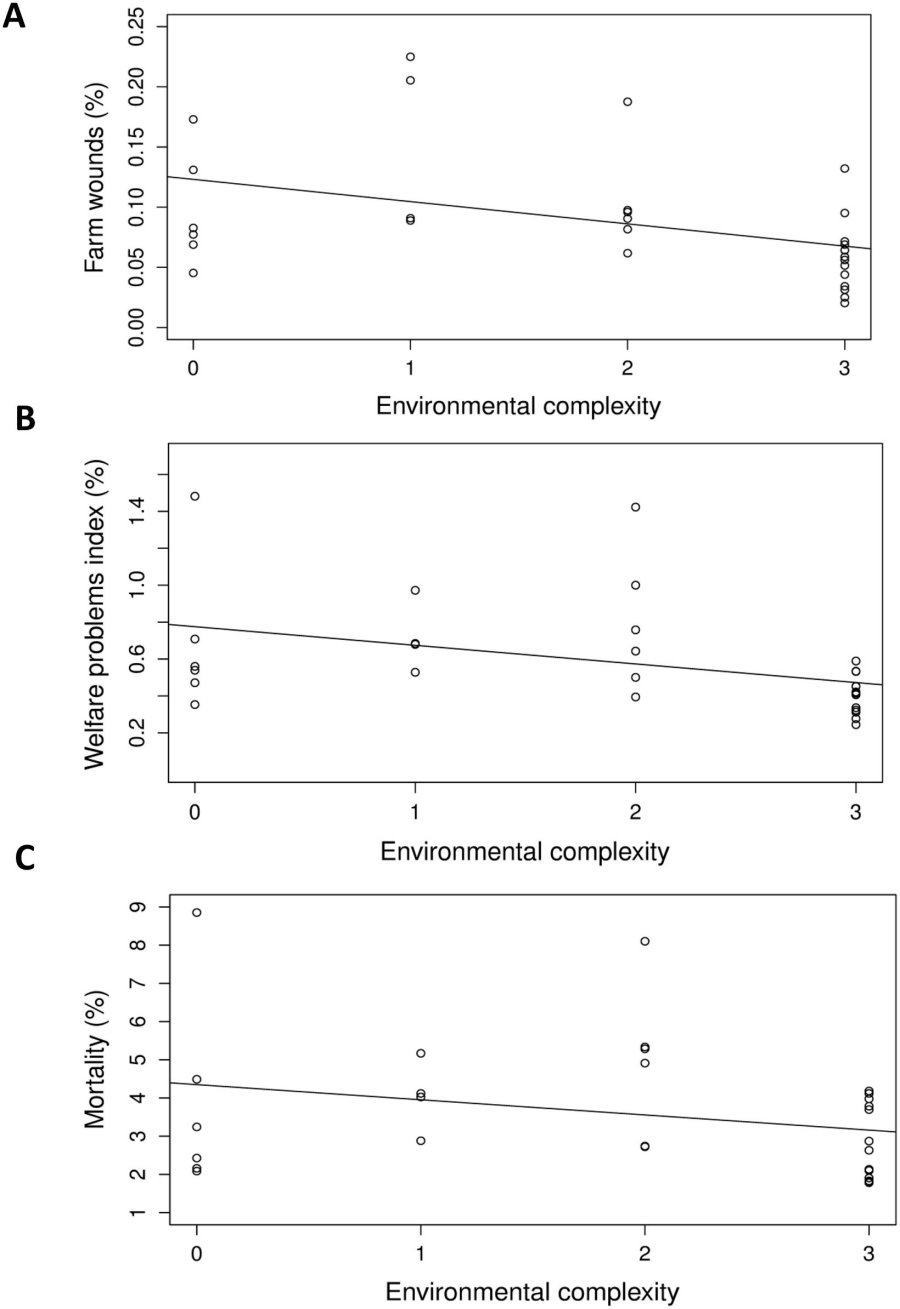
Associations of environmental complexity with (A) skin wounds (r^2^ = 0.183), (B) welfare problems index (r^2^ = 0.164), and (C) mortality (r^2^ = 0.074). Environmental complexity is based on the number of environmental enrichment types (boxes, peat, wood shavings bales) provided (from 0–3). Data points are back-transformed estimates. (A, B) Birds detected with skin wounds, and sum of birds detected with welfare problems (walking difficulties, illness, skin wounds, small size), as a % of the estimated number of birds in 4 assessed transects. (C) Number found dead and culled on the farm up to the day of slaughter as a % of number of chicks started.

**Fig 2:**
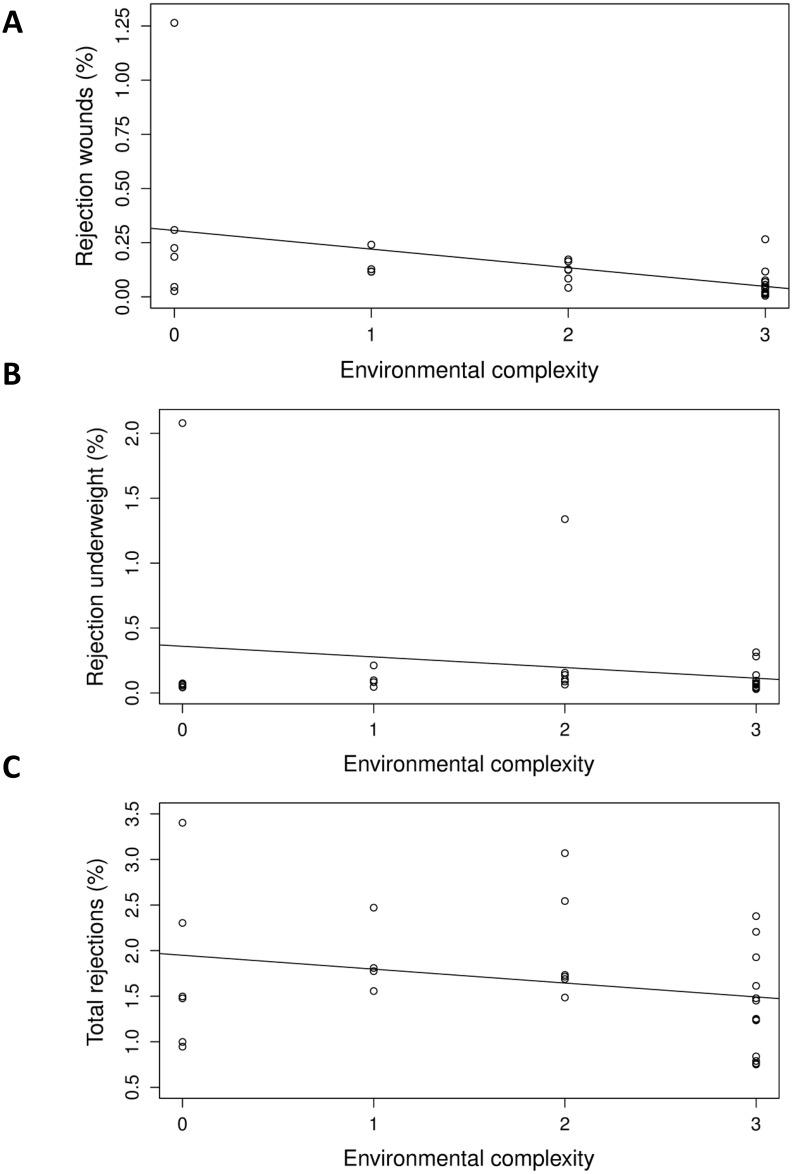
Associations of environmental complexity with rejections due to (A) wounds (r^2^ = 0.206), and (B) underweight birds (r^2^ = 0.053), and (C) total rejections (r^2^ = 0.076). Environmental complexity is based on the number of environmental enrichment types (boxes, peat, wood shavings bales) provided (from 0–3). Data points are back-transformed estimates. (A, B, C) Carcasses rejected as a % of total number of slaughtered birds.

With increasing environmental complexity (Table 4), we detected fewer birds with skin wounds during the transect walks (P = 0.004; Fig 1A), and the overall welfare problems index was lower (P = 0.002; Fig 1B). Increasing environmental complexity was associated with lower mortality (P < 0.001; Fig 1C), a lower proportion of rejections due to wounds (P < 0.001; Fig 2A) and underweight birds (P = 0.002; Fig 2B), and a lower overall rejection rate (P < 0.001; Fig 2C).

**Table 4:** Regression coefficient estimates for associations of environmental complexity and space allowance with welfare problems detected by transect sampling, and production outcomes.

Variable	Environmental complexity^1^						Residuals of space allowance^2^					
	Mean	SE	Lower 95% CL	Upper 95% CL	F^3^	P	Mean	SE	Lower 95% CL	Upper 95% CL	F^3^	P
***On-farm welfare assessment indicators***												
Walking difficulties	-0.09	0.04	-0.18	-0.01	4.50	0.055	-57.95	11.09	-82.11	-33.79	27.31	<0.001
Illness	-0.04	0.08	-0.20	0.13	0.24	0.632	5.77	19.48	-36.66	48.21	0.09	0.772
Skin wounds	-0.26	0.08	-0.43	-0.10	12.46	0.004	-31.27	20.41	-75.73	13.19	2.35	0.151
Small	-0.09	0.07	-0.23	0.05	1.94	0.189	34.11	17.25	-3.49	71.70	3.91	0.072
Welfare problems index	-0.12	0.03	-0.19	-0.06	16.29	0.002	-26.79	7.93	-44.07	-9.51	11.41	0.006
Litter score	-0.09	0.05	-0.20	0.03	2.62	0.131	3.16	14.32	-28.03	34.35	0.05	0.829
***Production outcomes***												
Mortality	-0.05	0.01	-0.06	-0.03	35.81	<0.001	1.09	1.91	-3.03	5.21	0.33	0.577
Rejection due to perosis	-0.19	0.15	-0.52	0.14	1.48	0.245	-56.67	38.87	-140.63	27.30	2.13	0.169
Rejection due to illness	0.03	0.01	<0.01	0.06	4.45	0.055	-2.92	3.50	-10.47	4.64	0.70	0.419
Rejection due to wounds	-0.35	0.04	-0.44	-0.27	88.99	<0.001	-78.88	13.51	-108.05	-49.70	34.11	<0.001
Rejection because underweight	-0.16	0.04	-0.25	-0.07	15.08	0.002	17.49	9.83	-3.74	38.72	3.17	0.099
Total rejections	-0.06	0.01	-0.08	-0.04	29.35	<0.001	-1.28	2.96	-7.66	5.11	0.19	0.673
Footpad score (binary)	0.10	0.34	-0.64	0.84	0.09	0.774	121.44	92.39	-78.15	321.03	1.73	0.211
Growth rate	-0.01	0.01	-0.02	0.01	2.17	0.164	4.89	1.50	1.65	8.12	10.63	0.006
Feed conversion	<0.01	0.01	-0.01	0.01	0.01	0.927	-1.64	1.45	-4.78	1.50	1.27	0.280
Production index	-0.01	0.01	-0.02	0.01	0.46	0.511	4.68	1.85	0.68	8.67	6.40	0.025

^1^Number of environmental enrichment types (boxes, peat, wood shavings bales) provided (0–3).

^2^Residuals of space allowance (m^2^/bird) regressed on environmental complexity.

^3^F_1, 12_ for welfare assessment variables; F_1, 13_ for production variables. See Methods and Table 3 footnotes for explanation of variables.

**Fig 3:**
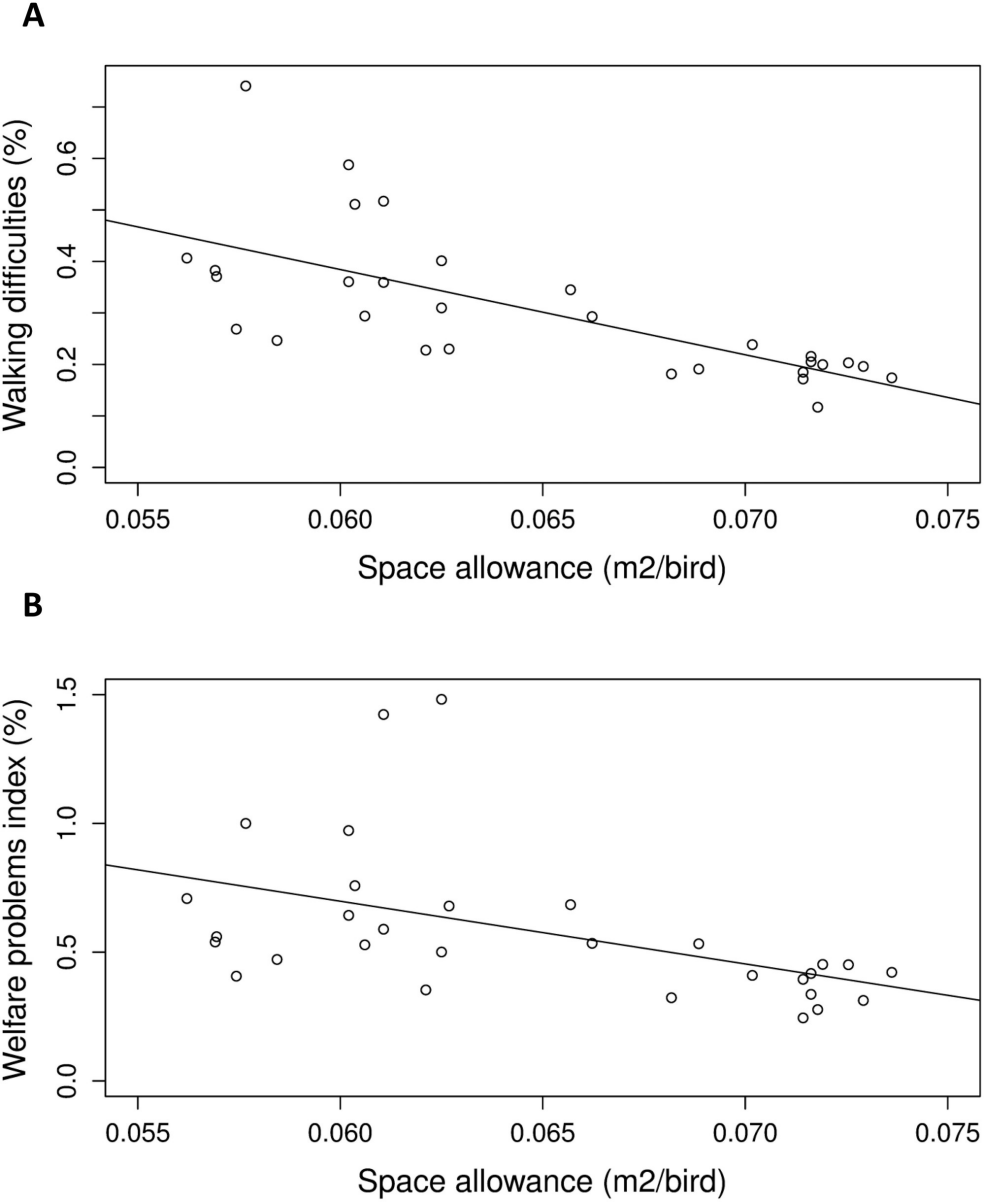
Associations of space allowance (m^2^/bird) with (A) walking difficulties (r^2^ = 0.480) and (B) welfare problems index (r^2^ = 0.233). Data points are back-transformed estimates from analysis of residuals of space allowance regressed on environmental complexity. (A) Birds detected with walking difficulties as a % of estimated number of birds in 4 assessed transects. (B) Sum of birds detected with welfare problems (walking difficulties, illness, skin wounds, small size), as a % of the estimated number of birds in 4 assessed transects.

**Fig 4:**
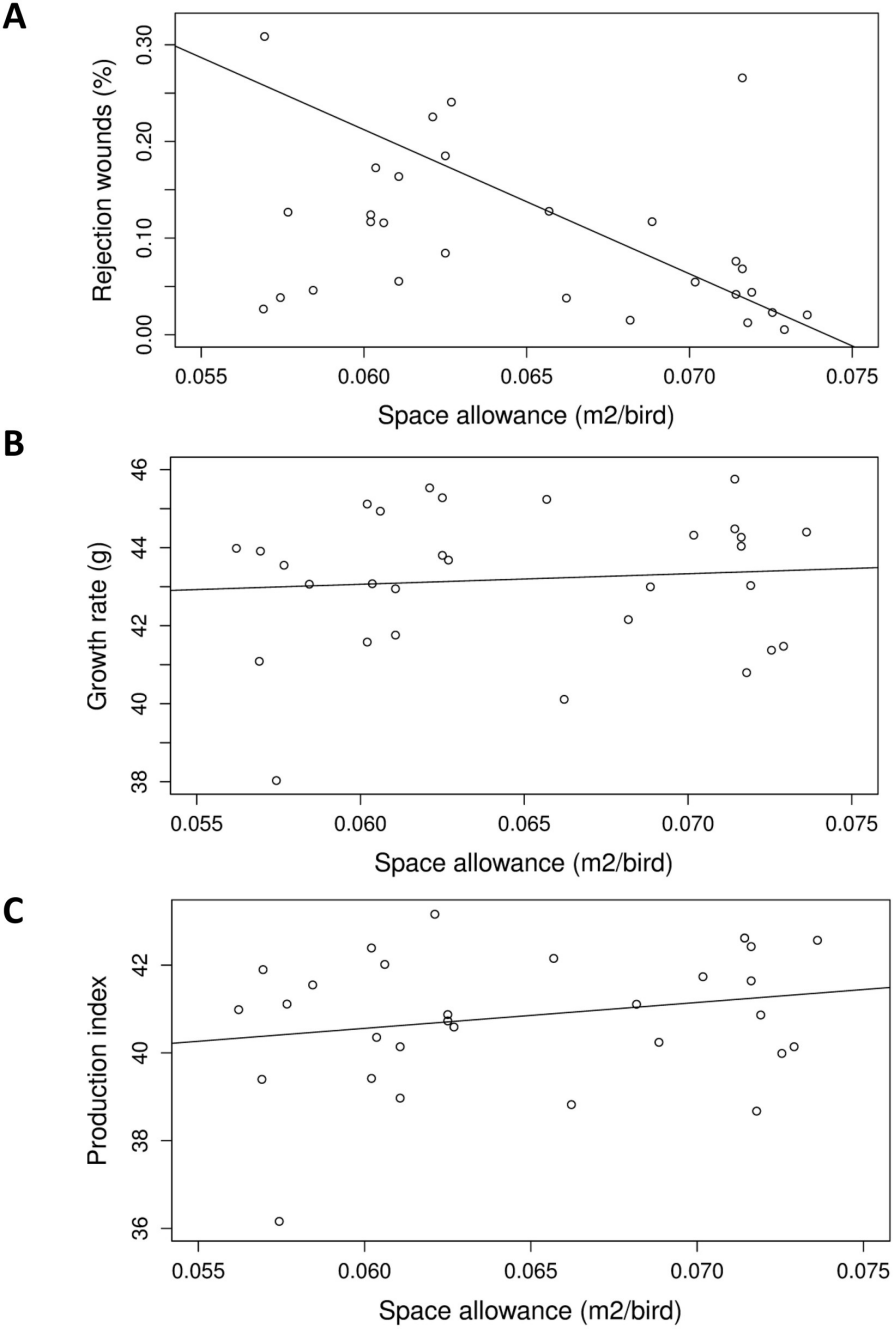
Associations of space allowance (m^2^/bird) with (A) rejections due to wounds (r^2^ = 0.151), (B) growth rate (r^2^ = 0.007), and (C) production index (r^2^ = 0.048). Data points are back-transformed estimates from analysis of residuals of space allowance regressed on environmental complexity. (A) Carcasses rejected due to wounds as a % of total slaughtered birds. (B) [Mean g eviscerated carcass weight/days of age at slaughter]. (C) [Mean g eviscerated carcass weight * (number slaughtered − number rejected)/(days of age at slaughter * number of chicks started)].

As space allowance residuals increased (Table 4), fewer birds with walking difficulties were detected (P < 0.001; Fig 3A) and the welfare problems index was lower (P = 0.006; Fig 3B). Higher space allowance residuals were also associated with a lower proportion of slaughter rejections due to wounds (P < 0.001; Fig 4A), a higher growth rate (P = 0.006; Fig 4B), and a higher production index overall (P = 0.025; Fig 4C).

The significant associations of environmental complexity and space allowance with welfare assessment findings and production outcomes had low to modest r^2^ values ranging from 0.007 to 0.480 (Figs 1–4).

**Fig 5:**
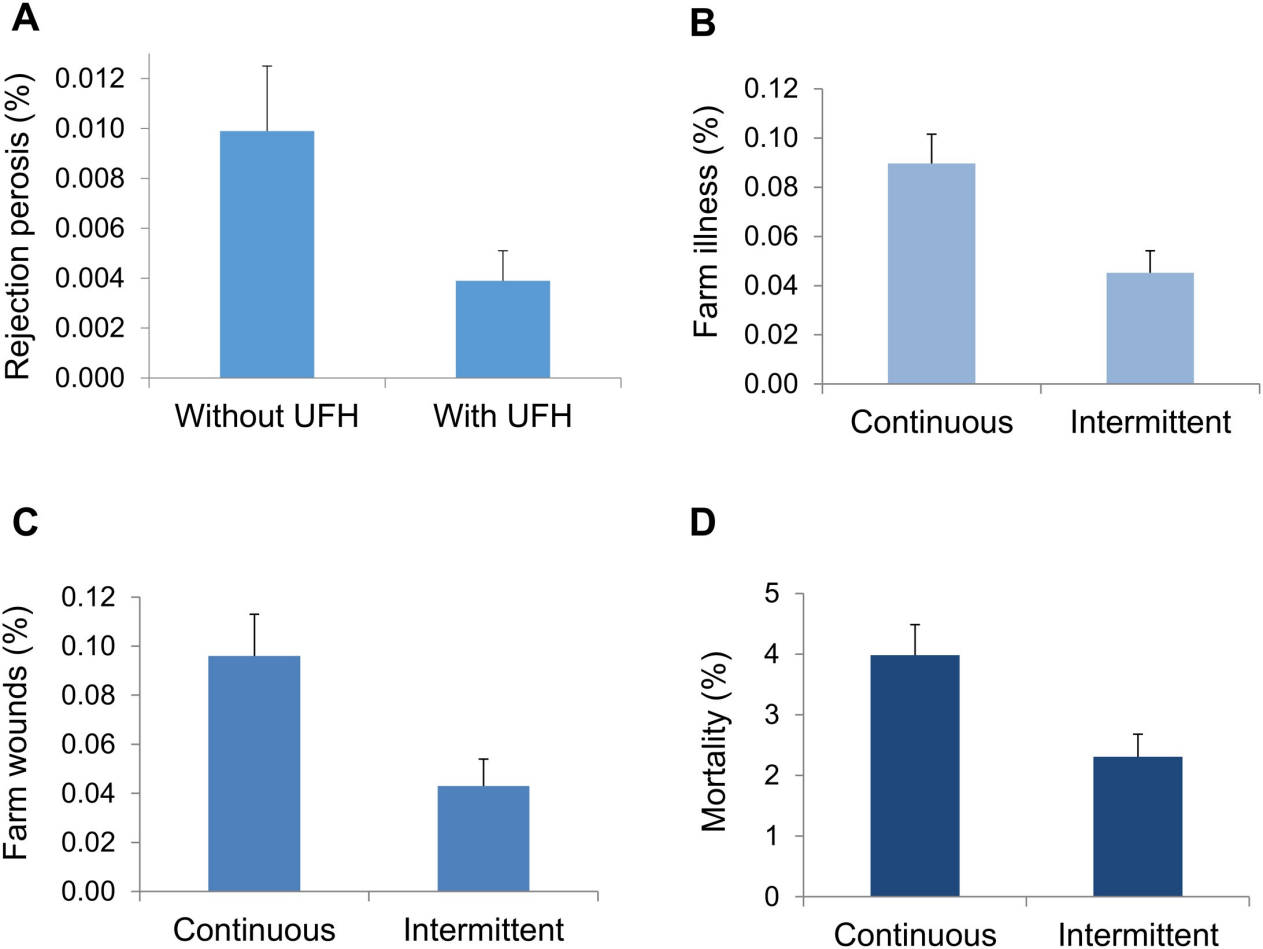
Associations of underfloor heating with (A) rejection due to perosis, and of photoperiod regimen on on-farm (B) illness, (C) skin wounds, and (D) mortality. Underfloor heating (UFH, without vs with). Photoperiod regimen (18 h continuous vs 16 h intermittent). Bars show back-transformed least squares means ± SE (differences, P < 0.05). (A) Carcasses rejected due to perosis (leg deformity) as a % of total number of slaughtered birds. (B, C) Birds detected with signs of illness, and skin wounds, as a % of the estimated number of birds in 4 assessed transects. (D) Number found dead and culled on the farm up to the day of slaughter as a % of number of chicks started.

The presence of underfloor heating (Table 5) was associated with fewer rejections due to perosis (P = 0.037; Fig 5A). The 16 h intermittent photoperiod regimen (Table 5) was associated with lower rates of illness (P = 0.015; Fig 5B) and skin wounds (P = 0.026; Fig 5C) on the farm than the 18 h continuous photoperiod regimen, as well as lower mortality (P = 0.022; Fig 5D).

**Table 5:** Back-transformed least squares means for associations of underfloor heating and photoperiod regimen with welfare problems detected by transect sampling and production outcomes.

Variable	Underfloor heating										Photoperiod regimen									
	Without				With				F^1^	P	18 h continuous				16 h intermittent				F^1^	P
	Mean	SE	Lower 95% CL	Upper 95% CL	Mean	SE	Lower 95% CL	Upper 95% CL			Mean	SE	Lower 95% CL	Upper 95% CL	Mean	SE	Lower 95% CL	Upper 95% CL		
***On-farm welfare assessment indicators***																				
Walking difficulties (%)	0.23	0.03	0.17	0.31	0.29	0.03	0.23	0.37	1.66	0.221	0.27	0.03	0.21	0.35	0.25	0.04	0.18	0.33	0.27	0.616
Illness (%)	0.08	0.01	0.05	0.12	0.05	0.01	0.04	0.07	3.62	0.081	0.09	0.01	0.07	0.12	0.05	0.01	0.03	0.07	8.11	0.015
Skin wounds (%)	0.07	0.02	0.04	0.12	0.06	0.01	0.04	0.09	0.14	0.717	0.10	0.02	0.07	0.14	0.04	0.01	0.03	0.08	6.47	0.026
Small (%)	0.05	0.02	0.02	0.10	0.10	0.03	0.05	0.17	2.22	0.162	0.08	0.02	0.04	0.14	0.06	0.02	0.03	0.13	0.18	0.678
Welfare problems index (%)	0.44	0.07	0.32	0.62	0.51	0.06	0.40	0.70	0.55	0.472	0.57	0.07	0.44	0.74	0.40	0.06	0.29	0.56	3.10	0.104
Litter score	1.34	0.16	1.02	1.75	1.08	0.11	0.87	1.34	1.72	0.214	1.13	0.11	0.91	1.41	1.27	0.16	0.97	1.66	0.44	0.518
***Production outcomes***																				
Mortality (%)	3.22	0.51	2.29	4.53	2.86	0.37	2.17	3.77	0.33	0.578	3.98	0.50	3.03	5.23	2.31	0.37	1.63	3.26	6.71	0.022
Rejection due to perosis (%)	0.01	<0.01	0.01	0.02	<0.01	<0.01	<0.01	0.01	5.41	0.037	0.01	<0.01	<0.01	0.01	0.01	<0.01	<0.01	0.01	0.15	0.709
Rejection due to illness (%)	1.34	0.21	0.96	1.87	0.98	0.12	0.75	1.28	2.33	0.151	1.25	0.16	0.96	1.64	1.05	0.16	0.75	1.46	0.77	0.397
Rejection due to wounds (%)	0.06	0.03	0.02	0.15	0.07	0.03	0.03	0.15	0.12	0.739	0.09	0.03	0.04	0.18	0.05	0.02	0.02	0.13	0.80	0.387
Rejection because underweight (%)	0.08	0.03	0.03	0.19	0.09	0.03	0.04	0.18	0.05	0.823	0.11	0.04	0.05	0.24	0.06	0.03	0.02	0.15	1.37	0.263
Total rejections (%)	1.65	0.26	1.17	2.33	1.35	0.17	1.02	1.78	0.92	0.355	1.79	0.23	1.36	2.35	1.25	0.20	0.88	1.77	2.86	0.115
Footpad score (binary)	0.39	0.17	0.13	0.75	0.38	0.13	0.16	0.68	<0.00	0.962	0.51	0.14	0.24	0.77	0.28	0.15	0.08	0.65	1.02	0.331
Growth rate (g/day)	42.18	0.82	40.44	44.00	43.68	0.69	42.22	45.19	1.80	0.203	42.86	0.68	41.43	44.35	42.99	0.84	41.21	44.84	0.01	0.914
Feed conversion (ratio)	2.27	0.04	2.19	2.35	2.21	0.03	2.14	2.27	1.71	0.214	2.23	0.03	2.16	2.29	2.25	0.04	2.17	2.33	0.18	0.676
Production index (g/day)	39.93	0.81	38.22	41.72	41.54	0.68	40.09	43.04	2.13	0.168	40.19	0.66	38.79	41.64	41.27	0.84	39.50	43.13	0.95	0.346

^1^F_1, 12_ for welfare assessment variables; F_1, 13_ for production variables. See Methods and Table 3 footnotes for explanation of variables.

### Relationships between welfare assessment findings and production outcomes

An increased prevalence of walking difficulties, illness and small size on the farm was associated with increased mortality, and increased rejections due to illness and underweight birds, and increased total rejections at slaughter (P < 0.05; Table 6). A higher prevalence of walking difficulties was also associated with increased rejections due to wounds (P < 0.001). A higher welfare problems index on the farm was associated with higher mortality, rejections in the illness, wounds, and underweight categories, and total rejections (P < 0.001). Higher litter scores were associated with lower mortality, but higher rejections due to illness, wounds and underweight birds, as well as total rejections (P < 0.01; Table 6). Litter scores were not associated with footpad dermatitis scores in this study.

**Table 6:** Regression coefficient estimates for relationships between welfare problems detected by transect sampling, and production outcomes.

		Production outcomes^1^									
On-farm welfare assessment indicators^2^		Mortality	Reasons for rejection				Total rejected	Footpad score	Growth rate	Feed conversion	Production index
			Perosis	Illness	Wounds	Under-weight					
Walking difficulties	Mean	162.53	254.75	38.82	457.45	146.61	78.23	-121.23	0.30	-4.44	-2.74
	SE	10.01	183.17	16.51	53.87	49.68	14.17	306.29	6.31	5.27	6.79
	Lower 95% CL	140.23	-153.39	2.04	337.43	35.92	46.66	-803.69	-13.77	-16.19	-17.86
	Upper 95% CL	184.83	662.88	75.59	577.48	257.30	109.80	561.23	14.36	7.31	12.39
	F_1, 10_	263.76	1.93	5.53	72.11	8.71	30.49	0.16	<0.01	0.71	0.16
	P	<0.001	0.195	0.041	<0.001	0.015	<0.001	0.701	0.963	0.419	0.696
Illness	Mean	-119.00	1085.85	804.52	-234.54	525.02	707.60	-267.01	-4.19	17.80	-18.88
	SE	45.82	596.08	71.29	234.15	200.61	62.55	1076.20	25.28	19.08	26.67
	Lower 95% CL	-221.09	-242.30	645.67	-756.25	78.04	568.24	-2664.94	-60.52	-24.70	-78.31
	Upper 95% CL	-16.92	2414.01	963.37	287.18	972.00	846.97	2130.92	52.15	60.30	40.56
	F_1, 10_	6.75	3.32	127.35	1.00	6.85	127.99	0.06	0.03	0.87	0.50
	P	0.027	0.099	<0.001	0.340	0.026	<0.001	0.809	0.872	0.373	0.495
Skin wounds	Mean	2.15	92.64	18.77	18.16	-74.41	56.61	1644.17	-0.43	-1.80	-0.90
	SE	21.08	384.19	32.20	105.85	125.56	28.33	890.20	15.76	13.44	17.03
	Lower 95% CL	-44.82	-763.39	-52.99	-217.68	-354.18	-6.52	-339.32	-35.55	-31.75	-38.84
	Upper 95% CL	49.11	948.66	90.52	254.00	205.36	119.73	3627.65	34.70	28.14	37.04
	F_1, 10_	0.01	0.06	0.34	0.03	0.35	3.99	3.41	<0.01	0.02	<0.01
	P	0.921	0.814	0.573	0.867	0.567	0.074	0.095	0.979	0.896	0.959
Small	Mean	148.00	-496.08	207.95	-5.35	312.85	237.95	227.03	2.77	-6.26	-6.93
	SE	10.54	398.05	18.86	59.43	39.10	14.56	421.99	5.59	3.77	5.78
	Lower 95% CL	124.51	-1382.98	165.92	-137.75	225.72	205.51	-713.22	-9.69	-14.67	-19.81
	Upper 95% CL	171.49	390.82	249.98	127.06	398.98	270.40	1167.28.95	15.23	2.15	5.96
	F_1, 10_	197.09	1.55	121.55	0.01	64.01	267.09	0.29	0.25	2.75	1.43
	P	<0.001	0.241	<0.001	0.930	<0.001	<0.001	0.602	0.631	0.128	0.259
Welfare problems index	Mean	118.19	79.18	98.53	243.75	266.67	135.85	184.61	0.78	-3.39	-6.051
	SE	5.74	63.11	11.01	21.17	28.26	8.74	138.77	3.37	2.40	3.56
	Lower 95% CL	105.78	-57.15	74.75	198.01	205.63	116.96	-115.17	-6.51	-8.57	-13.75
	Upper 95% CL	130.60	215.51	122.31	289.49	327.72	154.73	484.40	8.06	1.79	1.65
	F_1, 13_	423.44	1.57	80.14	132.53	89.07	241.48	1.77	0.05	2.00	2.88
	P	<0.001	0.232	<0.001	<0.001	<0.001	<0.001	0.206	0.822	0.181	0.113
Litter score	Mean	-0.16	1.47	0.36	1.62	0.59	0.50	-1.87	0.03	<0.01	0.02
	SE	0.04	0.82	0.06	0.25	0.17	0.06	1.42	0.03	0.02	0.03
	Lower 95% CL	-0.24	-0.31	0.22	1.08	0.22	0.38	-4.93	-0.03	-0.05	-0.05
	Upper 95% CL	-0.08	3.25	0.50	2.16	0.96	0.62	1.19	0.08	0.05	0.08
	F_1, 13_	18.25	3.19	31.70	42.61	12.05	76.19	1.75	0.93	<0.01	0.28
	P	<0.001	0.097	<0.001	<0.001	0.004	<0.001	0.209	0.353	0.988	0.605

^1^See Methods and Table 3 footnotes for explanation of variables.

^2^Walking difficulties, illness, skin wounds and small were predictors in one model, and the welfare problems index and litter score were predictors in separate models.

### Consistency between observers and transect locations

There was no difference between observers in the recording of each individual welfare indicator, but observers differed in the overall number of welfare indicators registered (P = 0.039; Table 7). A higher prevalence of skin wounds was detected on the right than left side of the house (P = 0.010). Illness, small size and welfare problems index values were higher in wall than central transects (P < 0.05). The total number of birds estimated to be present in the observed transects did not differ between observers, left vs right side, or wall vs central transects.

**Table 7:** Mean differences in prevalence of welfare problems (%) between transects according to observer identity and transect location (left minus right; wall minus central), with Wilcoxon signed-ranks test (S) results.

Comparison		Walking difficulties	Illness	Skin wounds	Small	Welfare problems index^1^	Birds (n)^2^
Observers	Mean	-0.04	-0.02	-0.03	-0.05	-0.14	-1.21
	SE	0.03	0.01	0.02	0.03	0.06	96.87
	S_n = 29_	-62.5	-71.5	-81.0	-62.0	-94.5	0.5
	P	0.181	0.124	0.064	0.162	0.039	0.992
Left vs right	Mean	-0.01	0.02	-0.04	0.01	-0.03	-35.84
	SE	0.03	0.01	0.02	0.03	0.06	96.63
	S_n = 29_	-15.5	37.5	-109.0	61.0	2.5	-85.5
	P	0.744	0.427	0.010	0.169	0.958	0.063
Wall vs central	Mean	0.04	0.04	-0.01	0.05	0.11	-78.27
	SE	0.03	0.02	0.01	0.02	0.05	215.19
	S_n = 29_	54.5	94.5	-22.0	116.0	96.5	-9.5
	P	0.245	0.039	0.625	0.006	0.034	0.841

^1^Difference in sum of individual welfare indicator counts as a % of the estimated number of birds in the observed transects.

^2^Difference in the estimated number of birds in the compared transects.

## Discussion

### Environment inputs

We expected the transect data to be positively associated with increasing environmental complexity based on previous reports suggesting beneficial effects when providing platforms, peat and bales of foraging material alone or in combination [16, 23, 32]. Indeed, we found that the overall welfare problems index declined with increasing environmental complexity, accompanied by reduced mortality and fewer rejections at slaughter due to wounds, underweight birds and overall. This might be because the enrichments stimulated multiple behavioural activities [33] having positive effects on health. We did not detect changes in growth rate, feed efficiency or production index value with increased environmental complexity. Although consumption of peat has previously been associated with increased weight gain and feed efficiency [29, 30, 31], the amounts provided in the current study were probably insufficient to affect flock growth. Also, provision of elevated structures such as perches and bales has not previously revealed effects on weight gain, feed conversion, or carcass yield [18, 58, 59].

The transect data revealed a reduced prevalence of skin wounds with increasing environmental complexity. The observed wounds were mainly scratches around the tail, most likely resulting from birds accidentally scratching one another with their claws when scrambling to avoid a perceived danger. The reduced skin wound rate is consistent with reports of reduced disturbances [60] and fear responses [61] in enriched houses, possibly influenced by more even distribution of birds within the house. Fewer rejections due to wounds suggest that experience with enrichments may have also resulted in calmer birds during catching. The non-significant tendency for reduced walking difficulties in enriched flocks (P < 0.10) is consistent with similar findings from other flocks provided with multiple types of enrichment under Norwegian housing conditions [33]. In Norway, farmers are required to promptly cull any birds that become immobile due to leg disorders. The lower mortality in flocks receiving more types of enrichments may, thus, be influenced by a beneficial effect of enrichment on leg health resulting in less culling.

Higher space allowance residuals at each level of environmental complexity were associated with fewer walking difficulties and welfare problems overall, as well as fewer rejections due to wounds and a higher growth rate and production index. These findings are consistent with previous studies reporting better gait scores with increased space allowance [34, 37, 62]. The improvement is possibly related to the impact of space on opportunities for locomotor activity [63], though greater space allowance does not always lead to increased use of space [64]. More space may facilitate access to feeders and drinkers, contributing to increased feed intake and weight gain with higher space allowance [39, 42]. Furthermore, improved walking ability with increased space allowance may have reduced the risk of injury during pre-slaughter handling [65], which could explain the reduced rejection rate due to wounds.

Collinearity between environmental complexity and space allowance raises the possibility that some results attributed to environmental complexity are at least partially explained by increased space allowance, particularly those in common with results based on the space allowance residuals (i.e. lower welfare problems index, fewer rejections due to wounds). However, differences in results for environmental complexity (i.e. lower mortality, fewer rejections of underweight birds and total rejections) and space allowance residuals (i.e. reduced walking difficulties and skin wounds, higher growth rate and production index) suggest that both factors make important contributions.

Although previous studies on underfloor heating are limited, beneficial effects have been reported in turkey and broilers [46, 47, 48]. In the current study, underfloor heating was associated with a reduction in rejections due to perosis (leg deformities), though these were infrequent (range 0–5 birds/flock). While underfloor heating affords a high degree of temperature uniformity throughout the house [43], other methods (e.g. use of heat exchangers [66]) can also be effective. All the houses in the current study were well insulated and equipped with modern automated heating, ventilation and drinker systems, explaining the relatively low litter scores, absence of dirty birds and lack of association between underfloor heating and footpad dermatitis scores. Underfloor heating also has the potential to produce dusty conditions contributing to health problems, but we found no evidence for increased illness due to underfloor heating in the current study.

Illness and skin wounds detected during transect walks, and total mortality, were lower under the 16 h intermittent photoperiod regimen compared to the 18 h continuous regimen. These findings could be related to the shorter overall duration of daily light exposure, given that long photoperiods have been linked to greater fear [67] and an increased risk of mortality due to metabolic and skeletal diseases [68, 69, 70, 71]. Further, our findings could be related to providing two daily dark periods instead of one. For example, if the birds under 16 h intermittent light were less hungry when the lights came on after the relatively short dark periods, they may have engaged in less scramble competition at the feeders, resulting in fewer skin scratches. Intermittent photoperiod regimens have been associated with a reduction in leg problems [53], higher body weight gain [72], and a lower prevalence of footpad dermatitis [73]. However, we did not detect such differences in this study, possibly because the two lighting regimens were more similar to each other than those compared in the previous studies.

### Estimating production outcomes from welfare assessment indicators

We found that a higher welfare problems index was associated with an increase in flock mortality, rejection due to illness, wounds, underweight birds, and total rejections. These results are consistent with previous reports on relationships between on-farm welfare and production outcomes [5, 13, 45, 74, 75]. Further, our results indicate that a higher prevalence of walking difficulties on the farm was related to increased rejections due to wounds at slaughter. This is possibly because birds with impaired walking ability were at greater risk of being trampled by conspecifics during pre-slaughter catching and loading [65]. Higher litter scores were associated with higher rejections due to illness, wounds, underweight birds, and total rejections, in keeping with previous reports demonstrating associations between litter quality, welfare issues and production outcomes [13, 45, 62]. It was unexpected to find that higher litter scores were associated with lower mortality. Perhaps the rate of culling was lower in flocks experiencing deteriorating litter conditions, resulting in lower mortality on the farm and a correspondingly higher rejection rate at slaughter.

The correspondence of illness, small birds and overall welfare problems on the farm with illness, underweight birds and total rejections at slaughter, respectively, supports the validity of transect sampling for anticipating relative rates of rejections. Walking difficulties on the farm did not forecast perosis rejections, probably because compromised walking can occur for reasons other than leg deformities [76]. Walking difficulties may have precipitated, or been precipitated by, other conditions, explaining associations with rejections due to illness and underweight birds. For example, lameness could cause difficulties in accessing feed and water, thereby increasing vulnerability to illness and impairing growth. Bacterial infections can also produce lameness [76]. The lack of correspondence between skin wounds on the farm and wounds detected at slaughter could be explained by the latter including injuries sustained during pre-slaughter handling. The absence of a relationship between litter scores and footpad dermatitis scores was probably due to the generally good litter quality in this study.

The on-farm welfare assessment indicators were not associated with a reduced growth rate or production index, or a higher feed conversion ratio. Nor were walking difficulties related to growth rate in this study. Culling of disabled birds and the relatively early slaughter age of Norwegian flocks may account for these findings. Considering that welfare problems tend to increase with age [13], detection of a higher welfare problems index at around 28 days of age could potentially forecast compromised growth and a loss of feed efficiency in flocks kept to greater ages.

### Consistency measures

Wilcoxon signed-rank tests showed consistency between observers in the recording of individual welfare indicators, but an observer effect on the overall welfare problems index. Because each observer sampled a different pair of transects, some of the variation between observers may be attributable to factors other than observer effects. When comparing between house sides (left vs right), results were consistent for all welfare indicators except skin wounds, for which higher levels were detected on the right than the left side of the house. This finding may have been related to bird reactivity to people entering the house, given that the house entrance was located on the right side of the house at a majority of farms. We also observed more illness, small birds, and overall welfare problems in wall than central transects. There are reports showing that broilers tend to sit by the house walls [77, 78], and that more dead birds are found by walls than in central transects [4, 79], consistent with our finding. Uneven distribution of welfare issues within the house highlights the value of sampling both central and wall transects, and transects on both sides of the house, to obtain a representative sample.

Some heterogeneity of results between observers and in different locations in the house suggests that the ease of implementing transect sampling comes at a cost of some loss in precision. Nevertheless, transect sampling allows rapid surveillance of large numbers of birds, providing the cost efficiency that is necessary for widespread implementation [80].

### Limitations and future directions

Bird movement and double counting might affect the reliability of transect sampling, especially in the presence of environmental enrichments that could alter bird movement patterns and distribution. Further research to assess the detectability and rate of repeated sampling of the same birds would be useful. The welfare indicators used in this study were focused on clearly evident health problems. Because health status does not inform about satisfaction of behavioural motivations [81, 82], consideration should be given to extending the transect sampling method to include behavioural indicators of positive welfare [83], facilitating inferences about welfare based on additional dimensions of animal welfare.

Caution is needed in interpreting the detected associations. We conducted 142 tests of significance, of which 43 were significant. At a 5% probability level, we can expect that approximately 7 of the significant findings represent false discoveries. Because animal welfare is affected by complex interactions between genetic background, environmental conditions and management [84], some of the detected associations between on-farm and slaughter variables may have occurred due to the mutual influence of other, unmeasured factors. Relatively weak r^2^ values point to the presence of unexplained variance and possible non-linear effects. Future studies with larger sample sizes would support investigation of non-linear effects as well as evaluation of the robustness of our results from variables with low prevalence and wide confidence intervals. There is also a need for a more quantitative measurement scale for environmental complexity that captures variation in types and amounts of enrichment materials supplied.

Farmers may benefit from implementing transect walks in all of their flocks to aid in benchmarking of welfare indicators in specific houses. Such data would provide a basis for comparing current flock findings with prior house, farm and regional averages, helping to identify the effects of making specific changes. Results rising above established targets could then trigger more detailed investigation to pinpoint causal factors. Since factors that predispose birds to develop welfare problems can continue to affect flock members after others have been culled, detecting emerging problems through transect sampling could spur timely interventions to improve welfare in the current flock. Future research is needed to evaluate the relative value of assessments conducted at different ages.

## Conclusions

We have demonstrated that data collected using the transect sampling method enabled detection of differences in broiler chicken welfare associated with differing environmental provisions. The transect data were also associated with rejection at slaughter and certain other productivity outcomes. Thus, we have established the utility of this approach for animal-based welfare assessment, which is a prerequisite to widespread adoption. Our results also suggest that flock welfare can be improved by providing multiple types of environmental enrichment, increased space allowance (over the range 0.056–0.073 m^2^/chick started), underfloor heating and an intermittent lighting programme with two 4-h dark periods. We conclude that the transect sampling approach offers a practical method for acquiring direct, quantitative data on the welfare of chickens on the farm, rather than relying solely on indirect assessment of the farmer’s adherence to engineering standards that, alone, cannot assure good welfare [1, 2, 84]. It also offers information about the current welfare status of a flock in a timely manner for enabling interventions instead of waiting for data generated at the end of the production cycle (e.g. footpad dermatitis at slaughter, which occurred too rarely to be informative in the current study). Overall, our results support the soundness of the transect sampling method as a practical tool for swiftly assessing welfare in large broiler flocks.

## Supporting information

S1 Appendix Statistical models, covariance estimates and dataset. (XLSX)
